# Efficacy of Pirtobrutinib Monotherapy in Treatment-Naïve Chronic Lymphocytic Leukemia: A Bayesian Network Meta-Analysis of Randomized Controlled Trials

**DOI:** 10.3390/cancers18040660

**Published:** 2026-02-18

**Authors:** Toby A. Eyre, Lisa M. Hess, Ehsan Masoudi, Min-Hua Jen, Sarang Abhyankar, Peita L. Graham-Clarke, Naleen Raj Bhandari, Peter Maguire, Katherine B. Winfree, Marsha Tracey, Kaisa-Leena Taipale, Matthew S. Davids

**Affiliations:** 1 Oxford University Hospitals NHS Trust, London OX3 9DU, UK; 2 Eli Lilly and Company, Indianapolis, IN 46285, USA; 3 Eli Lilly and Company, 61352 Bad Hamburg, Germany; 4 Eli Lilly and Company, Basingstoke RG21 4FA, UK; 5 Eli Lilly and Company, Sydney 2000, Australia; 6 Eli Lilly and Company, T4D KD39 Cork, Ireland; 7 Dana Farber Cancer Institute, Boston, MA 02215, USA

**Keywords:** chronic lymphocytic leukemia (CLL), network meta-analysis, indirect comparison, Bayesian analysis, first-line therapy, treatment naïve, BTKi therapy

## Abstract

Background: There are multiple effective treatment options for patients diagnosed with chronic lymphocytic leukemia and small lymphocytic lymphoma (hereafter, simply CLL). In 2025, two phase 3 randomized clinical trials of pirtobrutinib, a non-covalent BTK inhibitor, were reported, demonstrating improved outcomes versus comparator therapies in the treatment-naïve setting (NCT05254743 and NCT05023980). Methods: A systematic literature review was conducted to identify RCTs in the first-line setting for CLL. A Bayesian NMA was performed to compare overall response rate (ORR) and progression-free survival (PFS) of pirtobrutinib versus treatments recommended by the National Comprehensive Cancer Network in the first-line setting, with a focus on BTKi monotherapy. Results: Eight unique trials were identified for comparison versus pirtobrutinib. Eligible RCTs formed two disconnected networks (pirtobrutinib, ibrutinib and zanubrutinib were in Network 1; acalabrutinib was in Network 2). Results from Network 1 for ORR showed an odds ratio (OR) = 0.56 (95% credible interval [CrI], 0.28, 1.12) for ibrutinib versus pirtobrutinib and OR = 0.50 (95% CrI, 0.20, 1.27) for zanubrutinib versus pirtobrutinib. The PFS of ibrutinib was inferior to pirtobrutinib (hazard ratio (HR) = 1.89, 95% CrI, 1.13, 3.19); the PFS HR comparing zanubrutinib with pirtobrutinib was 1.51 (95% CrI, 0.84, 2.72). Conclusions: This NMA shows that pirtobrutinib has better PFS outcomes than ibrutinib. While PFS outcomes suggest that pirtobrutinib is comparable to second-generation covalent BTKi monotherapies, uncertainty exists in the interpretation of the treatment effect, as evidenced by wide credible intervals. These findings suggest the value of pirtobrutinib as a future treatment option for patients in the first-line setting.

## Simple Summary

There are multiple treatments available for chronic lymphocytic leukemia (CLL). This network meta-analysis compared NCCN-recommended therapies in the first-line setting for patients with CLL, with a focus on Bruton tyrosine kinase inhibitor (BTKi) therapy. The results show that pirtobrutinib, a non-covalent BTK inhibitor, has a high probability of being the optimally ranked BTKi therapy in the first-line setting. The findings demonstrate that pirtobrutinib has better progression-free survival (PFS) outcomes than ibrutinib. While PFS outcomes suggest that pirtobrutinib is comparable to second-generation covalent BTKi monotherapies, uncertainty exists in the interpretation of the treatment effect, as evidenced by wide credible intervals. These findings suggest the value of pirtobrutinib as a future treatment option for patients in the first-line setting.

## 1. Introduction

Based on Surveillance, Epidemiology and Endpoint Results (SEERs) data, approximately 23,690 patients are diagnosed with chronic lymphocytic leukemia or small lymphocytic lymphoma (hereafter, simply CLL) each year [1,2]. Patient care has significantly evolved over the last decade with the addition of targeted therapies for the care of patients diagnosed with CLL [3,4,5,6]. Current National Comprehensive Cancer Network (NCCN) treatment guidelines (version 1.2026) recommend continuous covalent Bruton tyrosine kinase inhibitor (cBTKi)-based therapy, time-limited B-cell lymphoma 2 (BCL2) inhibitor combined with anti-CD20 therapy, or time-limited combination therapy of BCL2i plus cBTKi, with or without anti-CD20 [7]. In the U.S., most patients currently receive cBTKi-based treatment in the first-line setting [4,6]. Over time, the utilization of ibrutinib (first-generation cBTKi) has declined; the majority of patients now receive second-generation agents, acalabrutinib or zanubrutinib [8]. There is little evidence differentiating the efficacy of various treatment choices given the number of agents available in the BTKi class and the lack of head-to-head clinical trials of pirtobrutinib versus these agents.

In December 2025, the non-covalent BTKi, pirtobrutinib, received full approval in the post-cBTKi setting, following its initial conditional approval by the U.S. Food and Drug Administration. This full approval was based on the positive results of the BRUIN CLL-321 phase 3 trial (NCT04666038), which demonstrated the superior efficacy of pirtobrutinib monotherapy versus investigator’s choice of idelalisib plus rituximab or bendamustine plus rituximab (BR) [9]. Recently, two additional phase 3 trials have demonstrated the efficacy of pirtobrutinib. BRUIN CLL-314 (NCT05254743) is a global phase 3 open-label randomized controlled trial (RCT) that compared pirtobrutinib monotherapy with ibrutinib monotherapy in the treatment-naïve and relapsed/refractory settings among patients with CLL without prior cBTKi exposure [10]. BRUIN CLL-314 met its primary endpoint of non-inferiority of overall response rate (ORR) in the intent-to-treat and relapsed/refractory populations. In exploratory analyses of patients who were treatment naïve, ORR was 92.9% (95% CI: 86.4–96.9) for pirtobrutinib versus 85.8% (95% CI: 78.0–91.7) for ibrutinib. While progression-free survival (PFS) was immature, the hazard ratio (HR) for those who were treatment naïve was 0.50 (95% CI: 0.23–1.08) versus ibrutinib. The second trial, BRUIN CLL-313 (NCT05023980), is a global phase 3 open-label RCT comparing pirtobrutinib monotherapy versus BR in the first-line setting. This study demonstrated superior progression-free survival (PFS) (hazard ratio [HR] = 0.199. 95% CI: 0.11, 0.37; *p* < 0.0001) and ORR versus BR (94.3%, 95% CI: 89.1–97.5 vs. 80.9% 95% CI: 73.4–87.0 [11]. While these two trials establish the efficacy of pirtobrutinib in the BTKi naïve setting, including in the first-line setting, there is no direct evidence available for pirtobrutinib against second-generation cBTKi agents (zanubrutinib and acalabrutinib), which are now more commonly used in the U.S.

To address this gap in knowledge, a Bayesian network meta-analysis (NMA) was conducted to compare the ORR and PFS of pirtobrutinib with other NCCN-recommended therapies in the first-line setting, with a focus on BTKi monotherapy.

## 2. Materials and Methods

### 2.1. Systematic Literature Review

This NMA was designed and conducted in accordance with the Preferred Reporting Items for Systematic reviews and Meta-Analyses (PRISMA) guidelines [12,13]. A systematic literature review was conducted using a pre-specified literature search strategy in Medline, EMBASE, and Evidence-based Medicine (EBM) reviews via the OVID platform, including a search of gray literature to identify available clinical data in the first-line setting (Supplemental Materials Table S1). The search strategy was conducted in September 2025. Abstracts from congresses (gray literature) were searched from January 2022 to September 2025. The literature search was broad to encompass a variety of research for patients with CLL. All studies (observational, randomized, single-arm) were included per these criteria: reported data from patients with CLL, included non-experimental interventions, reported clinical efficacy outcomes, was a research publication (not a review or editorial), included at least 10 study participants, was not a trial in progress report, and was in the English language. Eligible studies for this meta-analysis included RCTs from those identified in the literature search where at least one of the treatment arms of the trial included a regimen listed in NCCN Guidelines (version 3.2025). At least one clinical outcome of ORR or PFS was required to be reported. Study selection followed a multiple-stage process: title and abstract screening, followed by full-text screening and eligibility assessment for this NMA. Two reviewers independently screened the identified records at each stage for eligibility until the final set of studies were defined. This set of studies was then evaluated among clinical experts to ensure no RCTs were missed in the evaluation. Upon identification of all eligible studies, additional hand searching was conducted to obtain all relevant publications to ensure all available data were extracted and available for analysis.

For each eligible study, two independent investigators extracted study details from the most recent publication of trial data, including baseline characteristics of patients, sample size, independent review committee (IRC)-assessed response rate (excluding partial response with lymphocytosis), and IRC-assessed PFS into a Microsoft Excel database. A third independent reviewer was involved in any case of discrepancies in the independent eligibility review or data extraction. Additionally, risk of bias was evaluated by two independent reviewers using the National Institute for Health and Care Excellence (NICE) methodology checklist for randomized controlled trials [14]. Data for pirtobrutinib were provided from the sponsor (Eli Lilly and Company) and validated by independent data extractors. The most recent publication/most mature data were used for each study in the primary analysis; however, additional publications were evaluated to obtain characteristics of the patient population (if missing in the most recent publication), and data from the first publication of each trial was also extracted for exploratory analysis purposes.

### 2.2. Statistical Analysis

Comparisons between pirtobrutinib and all treatment arms in eligible RCTs were conducted through a Bayesian mixed treatment comparison incorporating both direct and indirect evidence, as available. Analyses were conducted in accordance with the NICE Decision Support Unit (DSU) Technical Support Documents (TSDs) [15]. For PFS, the approach described by Woods et al. [16] was used to account for correlations in relative treatment effect estimates (HRs) arising from multi-arm trials. As per NICE DSU TSD 2, ref. [15] observed log HRs were included in the model through a normal likelihood. A binomial logistic meta-regression model was used to fit the response to treatment, using a binomial distribution with a logistic link function. The primary analysis used a random effects model; fixed effects models were also conducted. The posterior distributions were summarized, using the median and the 2.5 and 97.5 percentiles to estimate hazard ratios (HRs), odds ratios (ORs), and 95% credible intervals (95% CrIs) of all pairwise comparisons. Markov Chain Monte Carlo (MCMC) simulations were run for 80,000 burn-in simulations and monitored for a further 480,000 simulations with a thinning rate of 24 for all outcomes. Non-informative prior distributions were used for the model parameters. For treatment effects, priors were set to N (0,100^2). For the random effects model, the prior distribution for the between-study variance was uniformly distributed as uniform (0,8). Missing values were not imputed, as only reported outcome data were used in the analysis.

Furthermore, the posterior distributions were used to estimate the probability that one treatment is better than another for ORR and PFS, respectively. This probability was calculated based on the proportion of MCMC cycles in which the specific treatment estimate is better than the comparator [15]. Based on the posterior distributions of each intervention, the probability for each treatment being ranked at a specific place (e.g., first, second, third) according to the outcome was estimated.

Surface under these cumulative ranking curves (SUCRA) and *p*-scores were used to provide treatment ranking [17,18]. SUCRA values reflect a treatment’s performance across all possible ranks, whereas *p*-scores derived from probability of being best (optimal) reflect only the likelihood of achieving first rank.

### 2.3. Model Fit and Diagnostic Assessment

Model fit was evaluated using Deviance Information Criterion (DIC), with lower DIC indicating better fit. When comparing two DIC values, a difference of five or more is regarded as a meaningful difference [19]. Convergence of chains was assessed by visual inspection of trace and autocorrelation plots and on the Gelman–Rubin–Brooks diagnostic (R < 1.2). In case of non-convergence or slow convergence, the calculations were repeated with the use of an informative prior and higher number of iterations, burn-in and thinning. Heterogeneity was assessed visually by inspecting the magnitude and variability of the study results within each forest plot where the point estimate size was made proportional to the size of the study. The difference between fixed and random effects in treatment estimates was also assessed by visual inspection; outcomes were expected to be similar if between-study variability was low.

If there was suspected heterogeneity, potentially confounding study characteristics (such as patient age, sex, or other differences in baseline characteristics) were investigated by presenting a forest plot sorted by the values of the characteristic.

Consistency was assessed by evaluating model DIC. A leverage plot was used to assess influential data points by plotting standardized deviance residuals against leverage. A posterior mean deviance contribution plot was used to compare study-specific contributions under the two models. For each treatment contrast, summary statistics were computed under both models, including the posterior mean, median, standard deviation, and 95% credible intervals. These estimates allowed comparison between the inconsistency model and the consistency model to assess whether differences between direct and indirect evidence suggest meaningful inconsistency.

### 2.4. Post Hoc Analyses

Post hoc analyses were implemented to connect the disconnected network of studies identified in the literature search. First, equivalence was assumed for BR to investigators’ choice of fludarabine–cyclophosphamide–rituximab (FCR)/BR to enable connection of networks via BR, as has been done in other NMAs [20]. Second, exploratory analyses were conducted using the earliest known published data from the trials identified in the literature review to evaluate the potential influence of data maturity on the comparative outcomes.

All primary and post hoc analyses were performed using the rjags package [21].

## 3. Results

### 3.1. Search Results

**Figure 1:**
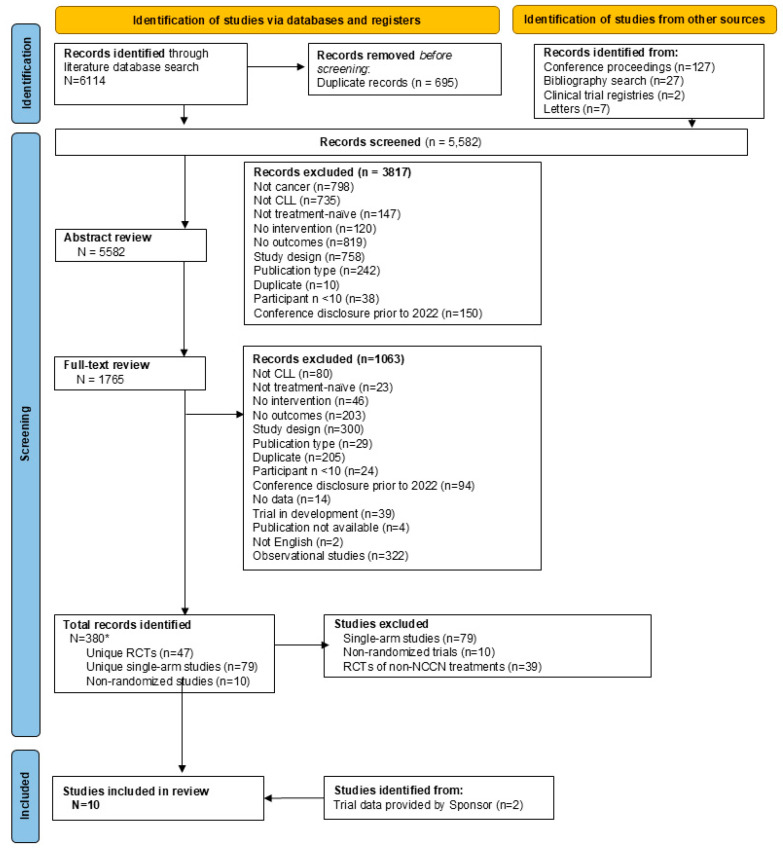
Screening and assessment of eligibility. CLL = chronic lymphocytic leukemia (inclusive of small lymphocytic leukemia); RCT = randomized controlled trial; NCCN = National Comprehensive Cancer Network. * Records were collapsed into studies at this point to ensure all data were captured for all eligible studies.

The initial systematic literature review identified 4259 unique publications. Additional updates, including hand searches, were conducted periodically through September 2025 to ensure that all available data were included in the NMA. Details of each step and reasons for exclusion are presented in Figure 1. A total of eight clinical trials were eligible for inclusion in this NMA for comparison against pirtobrutinib, which was reported in two clinical trials (BRUIN CLL-314 [NCT05254743] and BRUIN CLL-313 [NCT05023980]).

### 3.2. Networks of Eligible Studies

Details of all eligible studies and associated publications used in this study are summarized in Table 1. In general, these studies represented the treatment-naïve CLL population, which is known to have older age at diagnosis and the majority are male. Two studies (CLL13 and AMPLIFY) [22,23,24] may have had slightly more favorable characteristics, with both a younger age at diagnosis and fewer risk factors (such as Del17p or TP53 mutations) than other eligible studies. Despite variation in these two characteristics across trials (due to differences in trial eligibility criteria), all other factors across trials appeared representative of patients diagnosed with CLL.

**Figure 2:**
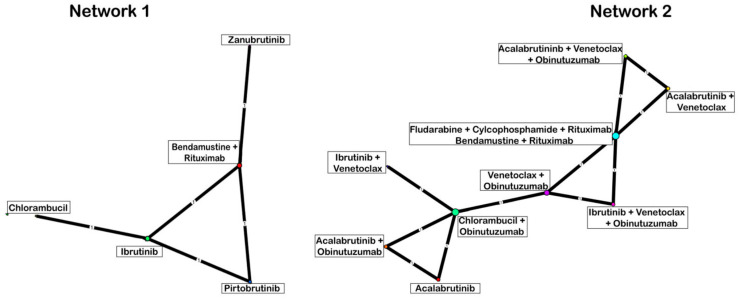
Networks of evidence.

Eligible studies formed two disconnected networks for ORR or PFS. The first network (Network 1) included pirtobrutinib, ibrutinib, and zanubrutinib, whereas the second network (Network 2) included acalabrutinib (Figure 2).

Risk of bias assessment is summarized in Table 2. Studies were generally of good quality, with lack of blinding (common in oncology) a consistent factor, leading to a high risk of performance bias across trials.

### 3.3. Model Fit and Diagnostic Assessment

All models demonstrated good convergence, and the network showed consistency (Table S2). Random effects models failed to converge, producing extremely wide and uninformative credible intervals. This reflects the small number of studies in each network, which is insufficient to reliably estimate between-study variance. The DIC showed minimal difference between models (difference <5; Table S2), and therefore fixed-effects models, as recommended by NICE DSU guidance, were used for all analyses.

### 3.4. Overall Response Rate: Network 1

**Figure 3:**
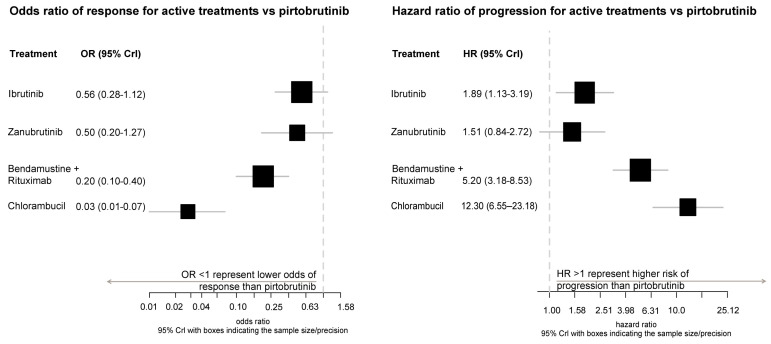
Overall response rate (**left**) and progression-free survival (**right**) of first-line treatments versus pirtobrutinib (Network 1).

ORR of ibrutinib versus pirtobrutinib showed an OR = 0.56 (CrI: 0.28–1.12) and zanubrutinib versus pirtobrutinib was OR = 0.50 (CrI: 0.20–1.27). Figure 3 presents the forest plots for ORR in Network 1. Pirtobrutinib had the highest probability of being the optimal treatment option for ORR in Network 1 (*p*-score = 0.891), followed by zanubrutinib (*p*-score = 0.067) and ibrutinib (*p*-score = 0.041). Table 3 provides a summary of *p*-scores and SUCRA values.

### 3.5. PFS of Network 1

PFS of ibrutinib versus pirtobrutinib showed a HR = 1.89 (CrI: 1.13–3.19), and for zanubrutinib vs. pirtobrutinib, HR = 1.51 (CrI: 0.84–2.72). Figure 3 presents the forest plots for PFS in Network 1. Pirtobrutinib had the highest probability of being the optimal treatment for PFS in Network 1 (*p*-score = 0.913), followed by zanubrutinib (*p*-score = 0.084) and ibrutinib (*p*-score = 0.003).

### 3.6. Post Hoc Analysis—Connected Network: ORR

**Figure 4:**
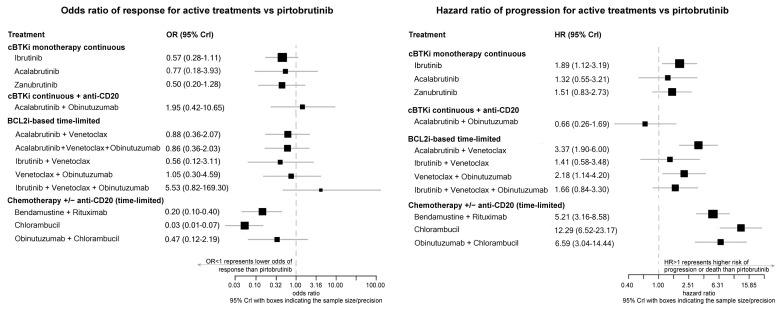
Overall response rate (**left**) and progression-free survival ^a^ (**Right**) of first-line treatments versus pirtobrutinib (connected network ^b^). ^a^ No HR was published for acalabrutinib–venetoclax–obinutuzumab; this comparator could not be included in the PFS analysis. ^b^ Assumes FCR/BR = BR [33] and preserves randomization. Abbreviations: cBTKi = covalent Bruton tyrosine kinase inhibitor; BCL2i = B-cell lymphoma 2 inhibitor; OR = odds ratio; CrI = credible interval; HR = hazard ratio.

Similar results were observed for BTKi agents versus pirtobrutinib when Network 1 was connected to Network 2. Outcomes for ibrutinib showed an OR = 0.57 (Crl: 0.28–1.11), acalabrutinib OR = 0.77 (Crl: 0.18–3.93), and for Zanubrutinib, OR = 0.50 (Crl: 0.20–1.28), all versus pirtobrutinib. Figure 4 presents the forest plots for ORR in the connected network for all treatment options compared to pirtobrutinib.

### 3.7. Post Hoc Analysis—Connected Network: PFS

Similar results to Network 1 were also observed for PFS between BTKi agents when the two networks were connected: acalabrutinib versus pirtobrutinib (HR = 1.32, Crl: 0.55–3.21); zanubrutinib versus pirtobrutinib (HR = 1.51, Crl: 0.83–2.73); and ibrutinib versus pirtobrutinib (HR = 1.89, Crl: 1.12–3.19). Figure 4 presents the forest plots of all comparators for PFS in the connected network.

Exploratory analyses using the earliest trial data published demonstrated consistent findings with the primary analysis (Supplementary Materials).

## 4. Discussion

In both Network 1 and the connected network, pirtobrutinib has the highest probability of being ranked as the optimal treatment option versus all BTKis for both outcomes, demonstrating an efficacy profile that is superior to ibrutinib for PFS, and is comparable to second-generation BTKi agents in the first-line setting for both ORR and PFS. Pirtobrutinib also shows comparable outcomes to time-limited combination therapies; however, data remain uncertain when making comparisons across the two networks. The efficacy results suggested by this NMA, combined with the known favorable toxicity profile of pirtobrutinib, provide additional confidence in the value that pirtobrutinib can add in the treatment-naïve setting, where treatment options are needed that not only provide efficacy, but also allow for choice and selection based on factors that matter to patients. These factors may be more relevant in first-line therapy (e.g., tolerability, ease of administration, duration of therapy) [34].

This NMA systematically reviewed the efficacy of treatments included in NCCN treatment guidelines to ensure relevance to health care decision makers in the U.S. Unlike a matching-adjusted indirect comparison (MAIC), NMAs preserve randomization inherent in each clinical trial. This enables more robust and unbiased comparisons across a network of randomized clinical trials through common comparators. Furthermore, NMAs allow for both direct and indirect comparison while generating a hierarchy of the treatment and comparators. As a result, NMAs are reproducible and widely accepted by national health care decision makers worldwide. The findings from Network 1 support the results from the BRUIN-CLL314 trial, which suggest favorable efficacy of pirtobrutinib versus ibrutinib [10]. These findings remain consistent in both direct and indirect evidence, supporting the overall interpretation of the findings from this network of evidence, and provide additional support for the consistency of comparable efficacy versus zanubrutinib observed in this study. Despite these strengths, the study does have several limitations.

Due to the low number of studies for each comparator and the uncertainty when connecting Network 1 to Network 2 (specifically related to the assumption of equivalence between BR and investigator’s choice of FCR or BR), this NMA is limited in its interpretation and cannot lead to any definitive conclusions regarding potential differentiation between pirtobrutinib and the second-generation cBTKi agent, acalabrutinib. While point estimates may suggest higher odds of response and lower risk of progression for pirtobrutinib versus zanubrutinib in Network 1, the credible intervals are wide and cross 1; results are not statistically different. Del17p and TP53 are important risk factors for CLL and were exclusion criteria for a number of trials included in this analysis. The heterogeneity of these risk factors from the included studies may have impacted the analyses and interpretation of this NMA, particularly across the two networks. Caution should be taken in drawing conclusions about interventions that are not in the same network due to these differences and the efficacy assumptions made to connect the two networks through BR. The duration of study follow-up may limit comparability and have an impact on the interpretation of results; at the time of this analysis, the data for pirtobrutinib showed the least amount of follow-up time among all trials, which could have affected the findings. Sensitivity analyses showed consistency of findings when using initial outcomes reported from the comparator trials, with slight movement of point estimates in the direction favoring pirtobrutinib. Lastly, no superiority or inferiority between therapies can be inferred from this analysis. Any suggestions of differences in efficacy are hypothesis-generating and would require an adequately powered head-to-head/randomized comparison.

## 5. Conclusions

The results of this Bayesian network meta-analysis of RCTs of patients with treatment-naïve CLL show pirtobrutinib has a high probability of being ranked as the optimal BTKi therapy in the first-line setting, with outcomes that suggest that pirtobrutinib has better PFS outcomes than ibrutinib and is comparable to second-generation cBTKi monotherapies. While data contain uncertainties due to the small number of trials, these findings suggest the value of pirtobrutinib as a future treatment option for patients in the first-line setting.

## Figures and Tables

**Table 1:** Characteristics of eligible studies.

	Arm 1	Arm 2	Arm 3	Del17(*p*), %	TP53, %	Unmutated IGHV, %	Male, %	Median Age, Years	Median Follow-Up, Months
				Arm 1/Arm 2/Arm 3					
**Network 1**									
BRUIN CLL 314/20030 [10]	Pirtobrutinib ^a^	Ibrutinib ^a^	--	11.7/13.6	11.6/20.2	58.6/58.1	60.7/62.8	67/67	22.4
BRUIN CLL 313/20023 [11]	Pirtobrutinib ^a^	Bendamustine + Rituximab (BR) ^a^		2.1/4.1	8.0/10.1	56.7/56.7	58.9/63.8	65/66	28
SEQUOIA [25,26]	Zanubrutinib ^a^	Bendamustine + Rituximab (BR) ^a^	--	0.1/0.0	6.5/5.8	53.4/53.0	63.9/60.5	70/70	61.2
Alliance A041202 [27,28]	Ibrutinib ^a^	BR ^b^	Ibrutinib + Rituximab ^c^	5.0/7.7	8.9/9.2	63.1/57.7	67.6/65.0	71/70	55.0
RESONATE-2 [29]	Ibrutinib ^a^	Chlorambucil (CLB) ^b^	--	0.0/0.0	8.9/3.2	57.4/58.2	64.7/60.9	73/72	88.5
**Network 2**									
GAIA-CLL13 [22]	Venetoclax ^d^ + Obinutuzumab ^b^	Ibrutinib ^h^ + Venetoclax ^d^ + Obinutuzumab ^b^	Fludarabine + Cyclophosphamide + Rituximab (FCR)/BR ^b^	0.0/0.0/0.0	0.0/0.0/0.0	57.0/53.2/57.2	74.7/68.4/71.2	62/60/61	38.8
AMPLIFY [23,24]	Acalabrutinib ^g^ + Venetoclax ^d^	Acalabrutinib ^g^ + Venetoclax ^d^ + Obinutuzumab ^b^	FCR/BR ^b^	0.0/0.0/0.0	0.0/0.0/0.0	57.4/59.1/59.3	61.2/69.2/63.1	61/61/61	40.8
ELEVATE-TN [30]	Acalabrutinib ^a^	Acalabrutinib ^a^ + Obinutuzumab ^e^	CLB ^b^ + Obinutuzumab ^e^	8.9/9.5/9.0	10.6/11.7	66.5/57.5/65.5	62.0/62.0/59.9	70/70/71	28.3
CLL14 [31]	Venetoclax ^d^ + Obinutuzumab ^b^	CLB ^d^ + Obinutuzumab ^b^	--	8.1/6.7	11.1/8.3	60.5/59.1	67.6/66.2	72/71	76.4
GLOW [32]	Ibrutninib ^f^ + Venetoclax ^d^	CLB + Obinutuzumab ^b^	--	0.0/0.0	6.6/1.9	67.7/62.0	55.7/60.0	71/71	46.0

^a^ Continuous therapy; ^b^ given for 6 cycles of therapy; ^c^ not included in analysis as not listed in guidelines, did not connect to other therapies; ^d^ 12-month time-limited therapy. ^e^ Given for 6 cycles of therapy; ^f^ 3-cycle lead-in followed by 12-month time-limited therapy; ^g^ 14-cycle time-limited therapy; ^h^ 36-month time-limited therapy.

**Table 2:** Risk of bias.

Trial	Selection Bias	Performance Bias	Attrition Bias	Detection Bias
BRUIN CLL314/20030	Low risk of bias	High risk of bias	Low risk of bias	Low risk of bias
BRUIN CLL313/20023	Low risk of bias	High risk of bias	Low risk of bias	Low risk of bias
SEQUOIA	Low risk of bias	High risk of bias	Low risk of bias	Low risk of bias
Alliance A041202	Unclear/unknown risk of bias	High risk of bias	Low risk of bias	Unclear/unknown risk of bias
RESONATE-2	Low risk of bias	High risk of bias	High risk of bias	Low risk of bias
GAIA–CLL13	Low risk of bias	High risk of bias	Low risk of bias	Unclear/unknown risk of bias
ELEVATE-TN	Low risk of bias	High risk of bias	Low risk of bias	High risk of bias
CLL14	Low risk of bias	High risk of bias	Low risk of bias	Unclear/unknown risk of bias
GLOW	Low risk of bias	High risk of bias	High risk of bias	Low risk of bias
AMPLIFY	Low risk of bias	High risk of bias	Low risk of bias	Low risk of bias

**Table 3:** SUCRA and *p*-score values.

Intervention	ORR Network 1		PFS Network 1		ORR Connected Network: Post Hoc ^a^		PFS Connected Network: Post Hoc ^a^	
	SUCRA	*p*-Score	SUCRA	*p*-Score	SUCRA	*p*-Score	SUCRA	*p*-Score
Pirtobrutinib	96.9%	0.891	97.7%	0.913	67.2%	0.015	86.5%	0.187
Ibrutinib	66.5%	0.041	54.3%	0.003	38.4%	0.000	51.2%	0.000
Acalabrutinib	---	---	---	---	54.6%	0.000	72.4%	0.003
Zanubrutinib	61.5%	0.067	73.0%	0.084	34.0%	0.000	66.5%	0.005
Acalabrutinib+ Obinutuzumab	---	---	---	---	87.7%	0.181	99.7%	0.795
Acalabrutinib + Venetoclax	---	---	---	---	61.0%	0.004	27.8%	0.000
Acalabrutinib + Venetoclax-Obinutuzumab	---	---	---	---	60.1%	0.004	---	---
Venetoclax + Obinutuzumab	---	---	---	---	69.5%	0.004	42.2%	0.000
Ibrutinib + Venetoclax	---	---	---	---	40.3%	0.001	68.5%	0.007
Ibrutinib + Venetoclax + Obinutuzumab	---	---	---	---	96.4%	0.790	59.9%	0.003
Bendamustine + Rituximab (BR)	25.0%	0.000	25.0%	0.000	9.9%	0.000	8.5%	0.000
Chlorambucil (CLB)	0.0%	0.000	0.0%	0.000	0.0%	0.000	0.0%	0.000
CLB + Obinutuzumab	---	---	---	---	30.9%	0.000	19.8%	0.000

^a^ Post hoc analyses (assuming FCR/BR = BR) were applied and preserves randomization. NOTE: SUCRA (surface under the cumulative ranking curve) and *p*-score values are scored from lowest to highest, reflecting the probability of being an optimal therapy.

## Data Availability

Eli Lilly provides access to all individual participant data collected during the trial, after anonymization, with the exception of pharmacokinetic or genetic data. Data are available to request 6 months after the indication studied has been approved in the USA and EU and after primary publication acceptance, whichever is later. No expiration date of data requests is currently set once data are made available. Access is provided after a proposal has been approved by an independent review committee identified for this purpose and after receipt of a signed data sharing agreement. Data and documents, including the study protocol, statistical analysis plan, clinical study report, and blank or annotated case report forms, will be provided in a secure data-sharing environment. For details on submitting a request, see the instructions provided at www.vivli.org.

## Supplementary Materials

The following supporting information can be downloaded at: https://www.mdpi.com/article/10.3390/cancers18040660/s1. Figure S1: Network 1-ORR SUCRA Plots; Figure S2: Network 1-PFS SUCRA Plots; Figure S3: Connected Network ORR SUCRA Plots; Figure S4: Connected Network PFS SUCRA Plots; Figure S5: Exploratory Analysis of ORR and PFS of First-line Treatments versus Pirtobrutinib (Network 1, first publication of trial data); Figure S6: Exploratory Analysis of ORR and PFS of First-line Treatments versus Pirtobrutinib (Connected Network^b^, first publication of trial data); Table S1: Medline search strategy applied in the systematic literature review; Table S2: Model fit.

## Abbreviations

The following abbreviations are used in this manuscript:

BCL2i	B-cell lymphoma 2 inhibitor
BR	Bendamustine + rituximab
BTKi	Bruton tyrosine kinase inhibitor
cBTKi	Covalent Bruton tyrosine kinase inhibitor
CI	Confidence interval
CLB	Chlorambucil
CLL	Chronic lymphocytic leukemia
CrI	Credible interval
DIC	Deviance Information Criterion
DSU	Decision Support Unit
EBM	Evidence-based Medicine
FCR	Fludarabine + cyclophosphamide + rituximab
HR	Hazard ratio
IRC	Independent review committee
MAIC	Matching-adjusted indirect comparison
MCMC	Markov Chain Monte Carlo
NCCN	National Comprehensive Cancer Network
NICE	National Institute for Health and Care Excellence
NMA	Network meta-analysis
OR	Odds ratio
ORR	Overall response rate
PFS	Progression-free survival
PRISMA	Preferred Reporting Items for Systematic reviews and Meta-Analyses
RCT	Randomized controlled trial
RE	Random effects
SLL	Small lymphocytic lymphoma
SUCRA	Surface under the cumulative ranking curve
