# Supplementary material for: Efficacy of Pirtobrutinib Monotherapy in Treatment-Naïve Chronic Lymphocytic Leukemia: A Bayesian Network Meta-Analysis of Randomized Controlled Trials
Source: Cancers (Basel). 2026 Feb 18;18(4):660. doi: 10.3390/cancers18040660 (PMC12939900; doi:10.3390/cancers18040660)
Supplement: Supplementary file 1 [file cancers-18-00660-s001.zip › cancers-4111755-supplementary.pdf]

cancers

MDPI

Article

Efficacy of pirtobrutinib monotherapy in treatment-naïve chronic lymphocytic leukemia: A Bayesian network meta-analysis of randomized controlled trials

Table S1. Medline search strategy applied in the systematic literature review

Step	Search strings
1	non-Hodgkin lymphoma/
2	exp chronic lymphatic leukemia/
3	(chronic lymphocytic leukemia or chronic lymphocytic leukaemia or chronic lymphatic leukemia or chronic lymphocytic or CLL).mp.
4	exp small lymphocytic lymphoma/
5	(small lymphocytic lymphoma or small-lymphocytic lymphoma or small lymphocytic or SLL).mp.
6	((chronic or small) adj3 (lymph* or leuk* or NHL)).mp.
7	or/1-6
8	(first line therapy or first-line or first line or 1st line or 1?st line or (first adj4 line) or frontline treatment or frontline therapy or untreated or initial treatment or treatment naive or treatment-naive or therapy naive or therapy-naive or treatment inexperienced or previously untreated or induction therapy or induction treatment or first-line to progression or first line to progression or newly diagnosed).mp.

9	7 and 8
10	Bruton Tyrosine Kinase inhibitor.mp.
11	(ibrutinib or imbruvica).mp.
12	acalabrutinib.mp.
13	(zanubrutinib or brukinsa).mp.
14	Tirabrutinib.mp.
15	(pirtobrutinib or LOXO-305 or "LOXO 305" or Loxo-305 or "Loxo 305").mp.
16	(BCL2* or BCL* or venetoclax or ABT*199 or (venetoclax adj3 therapy) or (venetoclax adj3 regimen)).mp.
17	exp Rituximab/
18	exp Alemtuzumab/
19	(Anti*CD20 or rituximab or obinutuzumab or alemtuzumab).mp.
20	exp Phosphoinositide-3 Kinase Inhibitors/
21	(Phosphoinositide 3*kinase inhibitors or PI3Ki or idelalisib or duvelisib).mp.
22	exp Immunotherapy/
23	(chemotherapy or chemotherapies or immunotherapy or immune therapy or chemoimmunotherapy or chemo-immunotherapy).mp.
24	or/10-23
25	9 and 24
26	exp adolescent/ or exp child/ or exp infant/ or (infant disease* or childhood disease*).ti,ab,kf. or (adolescen* or babies or baby or boy? or boyhood or child* or girl? or infant* or juvenil* or kid? or minors or minors* or neonat* or newborn* or new-born* or paediatric* or peadiatric* or pediatric* or perinat* or preschool* or puber* or pubescen* or school* or teen* or toddler? or underage? or under-age? or youth*).ti,ab,kf.
27	exp Case report/ or exp letter/
28	(Comment or Letter or Editorial or Note or Case Report or Case Study or Narrative Review or Practice Guideline).pt.
29	(nonhuman or animal experiment or animal tissue or animal cell or animal model or in vitro study or in vitro or in vitro studies or in vitro technique or in vitro techniques).mp.
30	or/26-29
31	25 not 30
32	limit 31 to (english language and humans)
33	limit 32 to yr="2010-Current"

Table S2. Model fit

Analysis	Model	Dbar	Dhat	pD	DIC
**ORR, Network 1**	RE	9.917438	0.039303	9.878135	19.79557
	FE	9.621745	0.52022	9.101525	18.72327
**PFS, Network 1**	RE	4.8299	0.001372	4.828528	9.658428
	FE	4.017099	0.034303	3.982796	7.999895
**ORR, Connected Network**	RE	23.15315	0.045321	23.10783	46.26099
	FE	22.90065	0.520289	22.38036	45.28102
**PFS, Connected Network**	RE	11.83839	0.001562	11.83682	23.67521
	FE	11.03448	0.034362	11.00012	22.03461

Abbreviations: FE=fixed effects; RE=random effects; Dbar=posterior mean residual deviance; Dhat=point estimate of deviance; pD=effective number of parameters; DIC=deviance information criterion

Surface under the cumulative ranking area (SUCRA) Plots

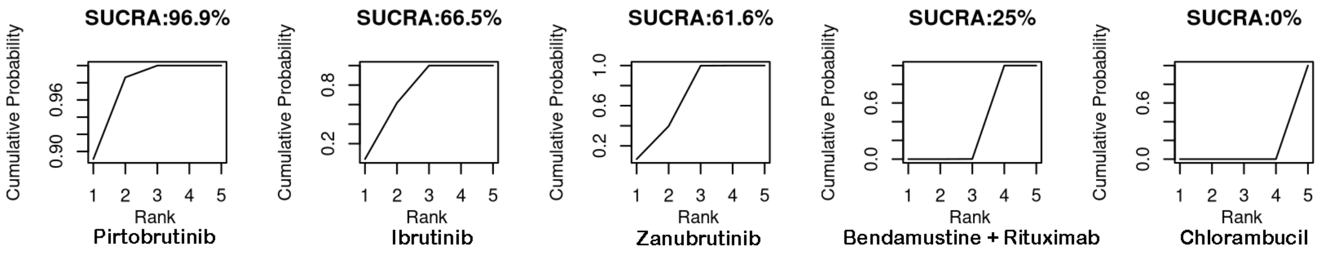

Figure S1. Network 1- ORR SUCRA Plots

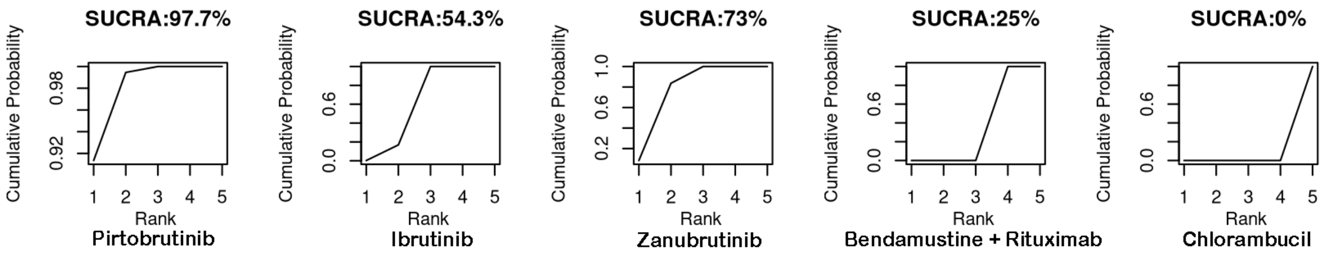

Figure S2. Network 1- PFS SUCRA Plots

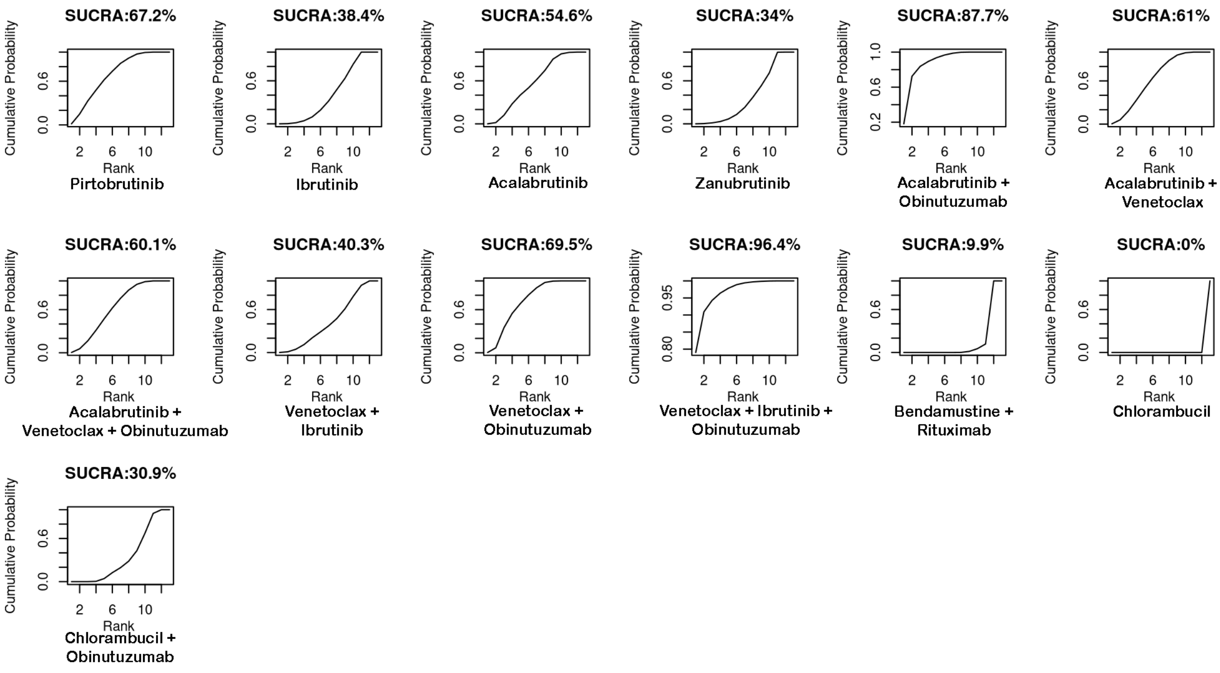

Figure S3. Connected Network- ORR SUCRA Plots

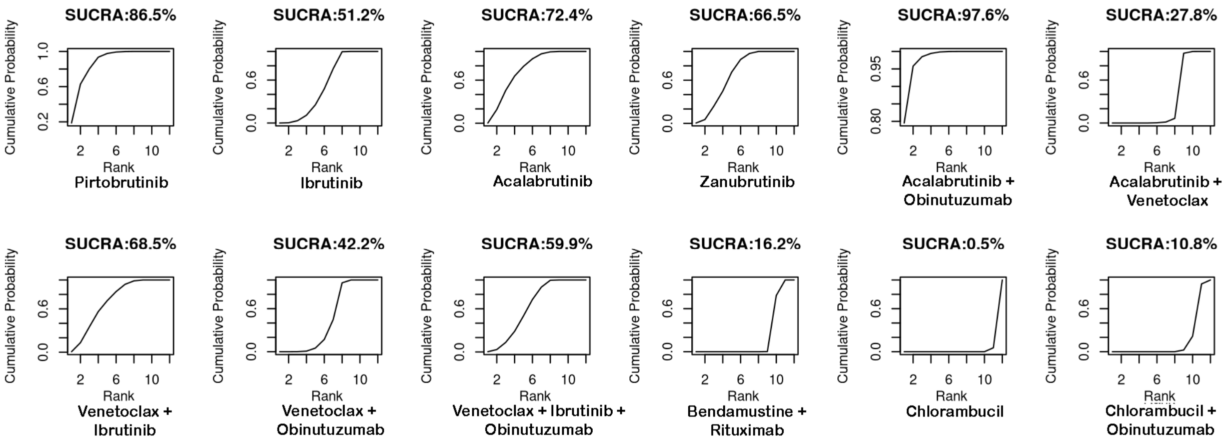

Figure S4. Connected Network- PFS SUCRA Plots

Exploratory analysis of first publications

This analysis evaluated the efficacy of active treatments versus pirtobrutinib using the first publication of trial data for each comparator in Network 1. As shown below, results are consistent with the primary analysis.

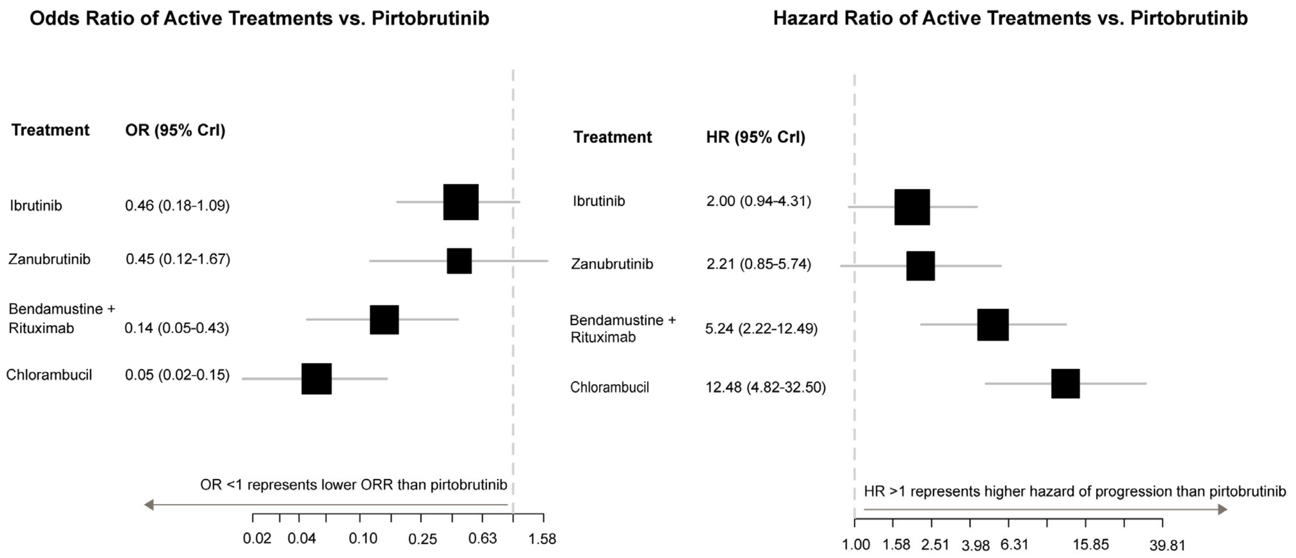

Figure S5. Exploratory Analysis of Overall response rate (left) and Progression-free Survival (right) of First-line Treatments versus Pirtobrutinib (Network 1, first publication of trial data)

Abbreviations: OR=odds ratio; CrI=credible interval; HR=hazard ratio.

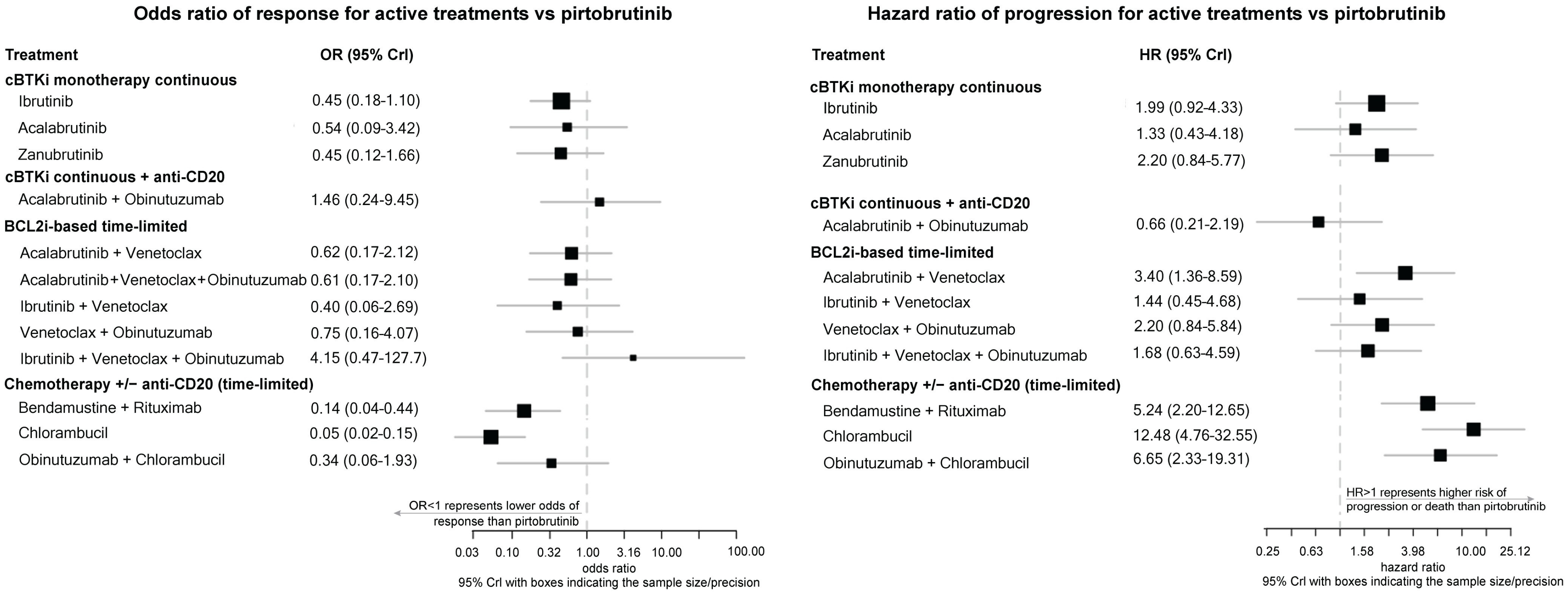

Figure S6. Exploratory Analysis of Overall Response Rate (left) and Progression-free Survival[a] (right) of First-line Treatments versus Pirtobrutinib (Connected Network[b], first publication of trial data)

[a]No HR was published for acalabrutinib+venetoclax+obinutuzumab; this comparator could not be included in the PFS analysis.

[b]Assumes FCR/BR = BR[37]and preserves randomization.

Abbreviations: cBTKi=covalent Bruton tyrosine kinase inhibitor; BCL2i=B-cell lymphoma 2 inhibitor; OR=odds ratio; CrI=credible interval; HR=hazard
